# Family physicians’ responses to personal protective equipment shortages in four regions in Canada: a qualitative study

**DOI:** 10.1186/s12875-022-01958-7

**Published:** 2023-02-27

**Authors:** Maria Mathews, Dana Ryan, Lindsay Hedden, Julia Lukewich, Emily Gard Marshall, Shabnam Asghari, Amanda Lee Terry, Richard Buote, Leslie Meredith, Lauren Moritz, Sarah Spencer, Judith B. Brown, Erin Christian, Thomas R. Freeman, Paul S. Gill, Shannon L. Sibbald, Eric Wong

**Affiliations:** 1 Department of Family Medicine, Schulich School of Medicine & Dentistry, Western University, 1151 Richmond Street, London, ON N6A 5C1 Canada; 2 Faculty of Health Sciences, Simon Fraser University, 8888 University Drive, Burnaby, BC V5A 1S6 Canada; 3 Faculty of Nursing, Memorial University, 300 Prince Philip Drive, St. John’s, NL A1B 3V6 Canada; 4 Department of Family Medicine Primary Care Research Unit, Dalhousie University, 1465 Brenton Street, Suite 402, Halifax, NS B3J 3T4 Canada; 5 Discipline of Family Medicine, Faculty of Medicine, Memorial University, 300 Prince Philip Drive, St. John’s, NL A1B 3V6 Canada; 6 Department of Epidemiology and Biostatistics, Schulich School of Medicine & Dentistry, Western University, 1151 Richmond Street, London, ON N6A 5C1 Canada; 7 Nova Scotia Health, 78 Lovett Lake Ct, Halifax, NS B3S 1B8 Canada; 8 Faculty of Health Sciences, Western University, 1151 Richmond Street, London, ON N6A 3K7 Canada; 9 Thames Valley Family Health Team, 1385 North Routledge Park, Unit 6, London, ON N6H 5N5 Canada; 10 St. Joseph’s Health Care London, Family Medical Centre, PO Box 5777, Stn B, London, ON N6A 4V2 Canada

**Keywords:** Primary care, Family physician, COVID-19, Pandemic response, Personal protective equipment, Policy planning, Qualitative research

## Abstract

**Background:**

Despite well-documented increased demands and shortages of personal protective equipment (PPE) during previous disease outbreaks, health systems in Canada were poorly prepared to meet the need for PPE during the COVID-19 pandemic. In the primary care sector, PPE shortages impacted the delivery of health services and contributed to increased workload, fear, and anxiety among primary care providers. This study examines family physicians’ (FPs) response to PPE shortages during the first year of the COVID-19 pandemic to inform future pandemic planning.

**Methods:**

As part of a multiple case study, we conducted semi-structured qualitative interviews with FPs across four regions in Canada. During the interviews, FPs were asked to describe the pandemic-related roles they performed over different stages of the pandemic, facilitators and barriers they experienced in performing these roles, and potential roles they could have filled. Interviews were transcribed and a thematic analysis approach was employed to identify recurring themes. For the current study, we examined themes related to PPE.

**Results:**

A total of 68 FPs were interviewed across the four regions. Four overarching themes were identified: 1) factors associated with good PPE access, 2) managing PPE shortages, 3) impact of PPE shortages on practice and providers, and 4) symbolism of PPE in primary care. There was a wide discrepancy in access to PPE both within and across regions, and integration with hospital or regional health authorities often resulted in better access than community-based practices. When PPE was limited, FPs described rationing and reusing these resources in an effort to conserve, which often resulted in anxiety and personal safety concerns. Many FPs expressed that PPE shortages had come to symbolize neglect and a lack of concern for the primary care sector in the pandemic response.

**Conclusions:**

During the COVID-19 pandemic response, hospital-centric plans and a lack of prioritization for primary care led to shortages of PPE for family physicians. This study highlights the need to consider primary care in PPE conservation and allocation strategies and to examine the influence of the underlying organization of primary care on PPE distribution during the pandemic.

**Supplementary Information:**

The online version contains supplementary material available at 10.1186/s12875-022-01958-7.

## Background

Despite the well-documented increase in demand, shortages of personal protective equipment (PPE) in previous disease outbreaks [1–3] and supply chain weaknesses that became apparent following Hurricanes Katrina and Maria [4], health systems were poorly prepared to meet the need for PPE such as gloves, gowns, masks, and face shields during the COVID-19 pandemic. Global competition, along with fragile supply chains and a desire to conserve limited supplies [5], contributed to PPE shortages in Canada during the COVID-19 pandemic [5]. Additionally, poor management resulted in provinces having absent or unreplenished stockpiles, despite recommendations from previous pandemic preparedness plans [6–8]. Regional entities responsible for managing PPE supplies chose to forego maintaining provincial stockpile warehouses, did not maintain adequate stockpiles for the scope and scale of the COVID-19 pandemic, did not have strategies to manage stockpile inventory (resulting in expired or outdated supplies), and failed to implement improvements to supply chain capacity that were recommended following the SARS and H1N1 outbreaks [7–9].

PPE shortages contributed to fear and anxiety in the health workforce, including among primary care providers [1, 3, 5, 10–15], which were compounded by the uncertainty about the epidemiology of the novel virus (e.g., mortality, symptoms, etc.), poor understanding of its transmission, frequent changes in protocols, and media reports from hard-hit regions in Italy, the United Kingdom, and the United States [2, 3, 5, 6, 8, 15].

PPE shortages impacted the delivery of health services and population health surveillance in Canada [4]. Specifically, PPE shortages in primary care led to reductions in the provision of preventative care [16–18], care for individuals experiencing marginalization [19], assessment and testing of patients with influenza-like illnesses [20], and in-person care [21]. We examine family physicians’ (FPs) response to PPE shortages during the first year of the COVID-19 pandemic to inform future pandemic planning. We describe factors related to access to PPE, strategies to mitigate PPE shortages, and the impact of PPE shortages on patient care and FP wellbeing. In Canada, while federal and provincial governments share responsibility for public health measures, provinces are responsible for delivering health services. This paper is part of a larger project examining FP roles during the COVID-19 pandemic, and is based on a multiple case study of four regions in Canada: the Vancouver Coastal health region in British Columbia (BC), the Eastern Health region of Newfoundland and Labrador (NL), the province of Nova Scotia (NS), and the Ontario (ON) Health West Region.

## Methods

As described in our published protocol [22], using a descriptive qualitative approach [23], we conducted semi-structured qualitative interviews with FPs from October 2020 to June 2021. Qualitative interviews are well-suited to understanding the context, constraints, and processes of behaviours from the perspectives of the participants [24]. Moreover, qualitative interviews are a flexible form of data collection that allowed us to adapt to the rapid changes in the provision of health care during the COVID-19 pandemic. We recruited FPs using maximum variation sampling [24] until we reached saturation (that is, until we had sufficient data to interpret the data rigorously and interviews revealed no new information on major themes) [24, 25]. We included FPs who were licensed to practice in 2020 and recruited along a wide range of characteristics to ensure we spoke with FPs with and without hospital and/or academic affiliations, different genders, varied practice models and payment types (e.g., fee-for-service, alternative payment plans, etc.), and different community sizes. Participants were eligible if they worked in primary care settings, including community family practice clinics, long-term care facilities, and hospitals. We excluded post-graduate medical trainees and FPs on temporary licenses or in exclusively academic, research, or administrative roles. In each region, research assistants emailed study invitations to FPs identified from faculty practice, team, and privileging lists, as well as the physician search portals of provincial medical regulators. We also posted recruitment notices in medical organisations’ newsletters, social media posts, and, where permitted by local ethics boards, used snowball sampling.

In each interview, we asked FPs to describe the various pandemic-related roles they performed over different stages of the pandemic and the facilitators and barriers they experienced in performing these roles, as well as other potential roles that FPs could have filled (Appendix A) [26]. We also gathered information about their background and practice characteristics. We tailored questions to account for regional differences in FP roles and health system contexts, including different pandemic responses. For example, we based subsequent questions and probes on FPs’ responses to questions about their practice model, payment, and nature of work which varied by region, individual preference, and location. Our questions also reflected the state of pandemic response at the time of the interviews (e.g., whether local hospitals were in crisis, introduction of vaccination, etc.). We conducted interviews by Zoom (Zoom Video Communications Inc.) or telephone, depending on participant preference. We audio-recorded interviews, which were then transcribed verbatim, and also included interviewer field notes in the analysis.

At least two members of the research team in each region read two to three transcripts independently to identify key words and codes, which were organized into a preliminary coding scheme, using a thematic analysis approach. To create a uniform coding template across the four regions, each regional team coded a set of four transcripts (one from each region) using their own coding template and then met to compare coding, refine the meaning of each code, and developed a unified template with consistent code labels and descriptions. One research assistant in each regional team used the unified coding template to code all transcripts and field notes using NVivo 12 (QSR International). We resolved any disagreement in coding through consensus. We summarized participant demographic and practice data using descriptive statistics. This paper examines the themes related to PPE.

We took several steps to ensure the rigour of our analyses [24, 25, 27], including pre-testing interview questions with FP and health system administrator team members, documenting procedures, using experienced interviewers, and verifying meaning with the participants during interviews. We looked for negative cases and provided thick description and illustrative quotes. Our research team included FPs and public health experts, allowing us to draw on prior expert knowledge in the development of our interview guide and the interpretation of our results [23].

We obtained approval from the research ethics boards in each region. Participants provided informed consent before interviews were scheduled. We reduced the risk of a privacy breach and maintained participant confidentiality through secure storage of recordings, password protection of electronic files, concealment of identifying information during the transcription process, and the use of study number codes to identify participants.

## Results

We interviewed a total of 68 FPs across the four regions (Table 1). Overall, the majority of participants were women (*n* = 41; 60.3%), had hospital privileges/affiliations (*n* = 49; 73.5%), and had their main practice setting in urban communities (*n* = 44; 64.7%). Participants described four overarching themes related to PPE: 1) factors associated with good PPE access, 2) managing PPE shortages, 3) impact of PPE shortages on practice and providers, and 4) symbolism of PPE in primary care.

**Table 1:** Characteristics of study participants by province

	Ontario ***n*** = 20 ***n*** (%)	Nova Scotia ***n*** = 21 ***n*** (%)	British Columbia ***n*** = 15 ***n*** (%)	Newfoundland & Labrador ***n*** = 12 ***n*** (%)	TOTAL ***n*** = 68 ***n*** (%)
Gender*					
Men	10 (50)	9 (42.9)	4 (36.4)	4 (33.3)	27 (39.7)
Women	10 (50)	12 (57.1)	11 (63.6)	8 (66.7)	41 (60.3)
**Practice Type**					
Fee-for Service	4 (20)	7 (33.3)	6 (40)	5 (41.7)	22 (32.4)
Alternative payment plan**	16 (80)	14 (66.7)	9 (60)	7 (58.3)	46 (67.6)
**Hospital Affiliation**					
No	15 (75)	6 (28.6)	3 (20)	5 (41.7)	18 (26.5)
Yes	5 (25)	15 (71.4)	12 (80)	7 (58.3)	49 (73.5)
**Community Size**^α^					
Rural	9 (45)	8 (38.1)	0 (0)	3 (25)	20 (29.4)
Small Urban	1 (5)	0 (0)	0 (0)	0 (0)	1 (1.5)
Urban	8 (40)	13 (61.9)	15 (100)	8 (66.7)	44 (64.7)
Mix	2 (10)	0 (0)	0 (0)	1 (8.3)	3 (4.4)
**Years in practice** (mean)	18.7	15.4	16.9	16.3	16.9

* Gender was asked as an open-ended question; ** Alternate payment includes all non-fee-for-service or enhanced fee-for-service payment types

α - Rural < 10,000 population, Small Urban =10,000–99,999 population, Urban > 100,0000 population

### Factors associated with good PPE access

In all four study regions, there was variable access to PPE among FPs:

*I’ve never had access to PPE issues* [BC05]

versus

*The first problem was the lack of PPE…* [BC10]

and

*we always had excellent access to any PPE that we needed* [NS02]

versus

*…we actually were worried about supply of PPE* [NS06].

In the first months of the pandemic, FPs whose clinics were based in hospitals, run by, or affiliated with a regional health authority or hospital generally enjoyed better and more stable access to PPE compared with community-based FPs:

*I think we were lucky in being in the Health Authority in that we didn’t have the same supply crunch that sort of, private practices did, like we, if we needed masks, we could get masks*. [BC12].

*… most of my work at the time was affiliated with [hospital], so we were reasonably well-stocked up through them… I did do some work in a walk-in clinic at that stage, where we just flat-out ran out of PPE*. [ON15].

Early in the pandemic, securing PPE entailed a lot of work:

*our supply chain for securing PPE was incredibly difficult, we had 3AM phone calls with ... Chinese suppliers… it was so much work to secure PPE …* [BC02].

Participants who had good access to PPE often belonged to teams with personnel dedicated to securing PPE:

*my clinic has a very excellent manager who has spent countless hours every week sourcing PPE from absolutely everywhere* [BC01]

and

*we had an excellent nurse who was very much on top of it… she was able to order adequate supplies to secure the necessary PPE for us, so we were very well, well-supplied, well-stocked...* [ON03].

### Managing PPE shortages

Participants who faced PPE shortages used many strategies to secure PPE. Some relied on home-made versions:

*I think we got like, a crazy number of face shields because there was a med student who was printing face shields using a 3D printer* [NL06]

and

*the community ended up sewing scrubs* [NS19].

Other participants received donations of PPE:

*In that first month of March [2020], we had a shortage of N95 masks, right. And the message went out in our community group here. And in that first couple of weeks, people were dropping off N95 masks. I had a guy who had N95 masks in their wood-shop drop off a box. I had two boxes dropped off at my desk without any question. I never asked for it. … We put out the word, you know, we were looking for scrubs. Literally the next day we had bags of scrubs show up.* [NS19].

With PPE back-ordered at regular medical suppliers, participants sought out PPE from hardware stores, often relying on products used in construction*: *

*I remember lining up at [name of hardware store] for two hours to try to get face shields for the clinic. … I remember looking on Amazon to buy like, shower caps to help supplement some sort of protection* [BC09]

and

*I honestly went online and I purchased a welding mask* [ON07].

Because of the concerns about running out of PPE, FPs often went to extremes to conserve PPE supplies, such as reusing masks:

*…we were putting a ‘Monday/Tuesday/Wednesday/Thursday/Friday’ on our masks, putting them in a paper bag and saving them for a week to put them on again, because we didn’t have enough PPE…* [BC11]

or

*using the same gown all day because we didn’t have adequate supplies* [ON12].

In many communities, centralized depots and distribution programs were organized. These programs initially began as community or medical student-led initiatives that were later taken over by regional health authorities and primary care networks, often with support from physician-led organizations. Participants in Ontario described how the local physician network organized PPE supplies for all health care workers in the region:

*The first major thing that we did was organizing for PPE. So, for PPE, a regional depot where we could mass order PPE and then physicians, dentists, pharmacists, any health professional who ran out of PPE could access an emergency supply through there.* [ON10].

Participants also described initiatives led by medical students in their region during the early stages of the pandemic:

*So, the medical student drive, I can’t tell you where they got the stuff, but they, I guess there was some businesses who … had stuff. … But they did a drive and collected it and then they distributed it to anybody who wanted it* [NL06].

In NL, as community-based physicians continued to experience PPE shortages, the regional health authority, with backing from the government and the medical association, organized a central depot. For some physicians, these depots worked well:

*…the RHA [regional health authority] had to supply us through drop-offs. …It went really well for us… Our staff went and picked it up and we never got less than what we asked for* [NL06]

while others expressed how the supply did not fully address their PPE needs:

…*they’d [community-based physicians] go to pick up the PPE and it was difficult to access because you had to go to a central place to get it and then they would request a certain amount of PPE and they’d come back to their office and it wouldn’t all be there, it would only be a smaller amount.* [NL04].

Similar centralized PPE depots available to community-based FPs were established in all four regions.

Securing PPE was also expensive:

*…there was price gouging. You know, when you see the mask cost at the beginning, and then the mask cost six months later, I think it was abhorrent how much companies were allowed to charge* [NS10]

and

*…once the closure hit and then when we started to order, the prices were like, already jacked up an enormous amount* [ON10].

A physician in NL noted that the added expense of PPE was borne by community-based physicians, but not by physicians whose PPE was provided by the regional health authority:

*there was a lot of discontent from other physicians about lack of PPE; I’m fortunate our clinic is funded by [the regional health authority], so we did have access to it, it wasn’t an issue and it wasn’t an issue of having to pay for it.* [NL04].

Some participants also worried about the quality of the PPE they received from new or unfamiliar suppliers:

*Sometimes I think we had poor quality stuff, …I can’t tell what level protection this is because it’s in a different language…. I’m not sure if this is really legitimate…* [NL06].

One participant described the efforts their practice went to be able to use otherwise faulty masks:

*we ended up buying all these masks through, I think from China through Amazon, and then all the strings would pull off every time you’d put them on your head. And so, I got nervous about the masks. … and then we got somebody – one of the other docs’ kid who came in and was paid per mask for gluing and he would glue like, 400 masks at a session….* [BC11].

Participants were not always assured that even high-quality masks fit appropriately because they may not have access to a consistent brand:

*There’s many different brands and versions of N95, and just because you might get mask fit-tested in Hamilton first, for instance for a particular mask, if you go out to do a rotation in Burlington, it might be a completely different mask… So, the mask fit-testing was actually not as helpful as it may have been in the past*. [ON17].

### Impact of PPE shortages on practice and providers

The shortages of PPE added to the sense of fear of COVID-19 among FPs, especially at the start of the pandemic when the transmission of the virus was poorly understood:

*…the disease being so new and us not really understanding it. I think the PPE issue added to that level of fear and feeling like we just didn’t have enough information or resources to manage the pandemic as it hit us* [ON12].

Participants noted that worrying about securing sufficient PPE and concerns about quality added to the emotional burden among primary care providers:

*There were times when PPE became pretty low and we weren’t sure how or where we would get it… and it was also a bit of a mental or emotional strain to be constantly trying to source PPE during that time* [BC01].

This emotional burden was exacerbated by

*the uncertainty and what seemed like a shortage in PPE…. I think we all sort of had, to a greater or lesser extent, angst around that* [NL01].

PPE was also a source of tension between practice staff and physicians, that was heightened by the rapidly evolving guidelines on appropriate infection prevention and control protocols:

*We didn’t know whether the virus was aerosolized or droplet at the time … And then [Public Health] kind of changed their minds and they decided it wasn’t aerosol, it was droplets, and trying to convince the nursing staff and physicians not to wear N95s and to wear surgical masks which were also coming in short supply - it was, it was awful.* [ON13].

One participant recalled having to explain to staff why they did not need the same level of PPE as physicians:

*So, dealing with the staff anxiety … Our front office staff felt that they all needed PPEs. Well, how do you convey to some of the fears in that early stage of needing PPE when in reality, if you’re not involved in aerosolizing generating procedures, that you don’t need a PPE? But in that early month, it was a difficult… because there was such uncertainty, right.* [NS19].

Working with PPE required additional time and changes to the workflow of their practices:

*Because the PPE required for donning and doffing … requires a lot of physical space and my clinic is small* [BC02]

and added to overall workload.

### Symbolism of PPE in primary care

Participants in the study also talked about the symbolic nature of PPE. One participant noted that the need to prioritize PPE for acute care centres was typical of the hospital-centric nature of the pandemic response:

*we initially had masks, but people were saying can you please give the masks to the hospitals? Which, again, was the wrong thing to be doing* [ON05].

A participant in BC felt that, even if they were performing a high-risk procedure, FPs in the community did not receive the same PPE protection as their hospital colleagues:

*And then especially for overdose prevention sites, where sometimes we were told, ‘oh you can probably bag mask ventilate with surgical masks’. And then in the ER, they were probably using N95s. So, I didn’t want to make this, like, hierarchy of PPE for community versus hospital* [BC02].

In NS, in the years preceding the pandemic, the government required family practices to have hospital privileges to integrate the primary care sector with the rest of the health system. However, one participant explained that these same family practices were not included in PPE considerations:

*But primary care seems to be separate from the hospitals. When we were asked to become part of [the health authority] and become part of the hospital …[but] when the issue of PPE came up, primary care was not even in the calculations of how much PPE was needed in the community* [NS07].

Similarly, in BC, a participant noted the difference in access to PPE when she took part in activities with the regional health authority and when she took part in the same activities through their private practice:

*And even just running a flu clinic…we were running some in collaboration with [the regional health authority], they provided syringes and needles, and full PPE for that if I do flu shots in their space. But if I do flu shots in my space, I don’t have access to any of that. I have to provide all of that. So, we’re in a very funny place as family physicians in the community where it sounds and it seems like we are all part of the process, all part of the pandemic care, yet we are completely left out of the supply chain for PPE and have been right from the start.* [BC01].

For many participants, the lack of concern about their poor PPE supply was symbolic of the lack of respect for primary care. In NL, where community-based physicians felt disrespected by the regional health authority’s inaction, one participant noted the affront of local fast-food workers having better access to PPE than community-based FPs:

*… [I had] the perpetual feeling of being disrespected because I was the one still out in the community with no PPE… [name of fast-food restaurant] had PPE. We didn’t have PPE. … And not to sound melodramatic, but that’s the truth of it. If you are seeing patients and you’ve got no PPE and you can go stop in and pick up a [fast-food] order and somebody’s got full PPE on, you know, that’s a kick in the guts, right?* [NL11].

## Discussion

During the early months of the COVID-19 pandemic, health systems across Canada prioritized hospital and hospital-affiliated sites for PPE, leaving FPs in independent, community-based practices scrambling to find appropriate PPE. Similar to other studies, we found that FPs rationed and reused PPE [5, 28] in an effort to conserve their available stocks. FPs also relied on donated and non-medical grade products, and PPE collection and redistribution efforts in their communities [3, 6, 29]. Concerns about shortages, reuse of PPE, and reliance on potentially sub-standard products contributed to anxiety and personal safety concerns, and created tensions between health professions [5, 8].

Integration with hospital or regional health authorities gave FPs better access to PPE (and at no additional cost or minimal administrative work compared to sourcing their own PPE), than FPs in community-based practices. These findings are consistent with reports that have documented the PPE conservation strategies that initially prioritized PPE for use in hospitals [5, 9] over community-based settings, including primary care. The hospital-centric approach illustrates the ongoing tension in the Canadian health system that strives to integrate primary care into the regional health care systems [30] while still viewing family practices as independent or private businesses [31]. Unsurprisingly, FPs who were part of large teams (the goals of recent primary care reform [30]) often enjoyed good access to PPE. In all four of the regions in our study, provincial governments (often in collaboration with regional health authorities and/or medical professional organizations) assumed responsibility for sourcing, storing, and distributing PPE supplies to primary care providers [32–35]. Preparations for similar centralized PPE distribution sites should become part of regional activities during the pre-pandemic period [26] when public health officials begin to warn FPs of potential pandemic-causing diseases. Moreover, responsible stewardship of PPE during the inter-pandemic period must include ensuring adequate supplies for primary care providers.

Medical masks have been associated with many social, cultural, and political meanings, especially in plagues and pandemics [36, 37]. During COVID-19, PPE held deep symbolic meaning for FPs, representing the health system’s lack of respect for primary care. The lack of priority placed on primary care in pandemic responses, including in the planning for PPE, has been reported in other high-income countries [38–41]. The limited representation of primary care leaders in pandemic response decision-making bodies [9, 42] contributed to hospital-centric PPE plans, and under-appreciated both the potential risk of COVID-19 to primary care providers [43], as well as the contribution of primary care in alleviating demand for hospital-based services [16].

Our findings are consistent with experiences of primary care providers in other countries. During the early stages of the COVID-19 pandemic, with the exception of Singapore [44], FPs around the world reported shortages of PPE [10, 11, 14, 45–47], but better access in hospital-affiliated clinics [3, 14, 40, 48]. FPs also re-used PPE, sourced PPE themselves, and used virtual care to reduce exposure risks and conserve PPE [3, 11, 12, 15, 46, 48, 49]. The risk of infection created anxiety for FPs who were particularly concerned about spreading COVID-19 to family members [3, 10, 12, 13, 15, 44, 48, 49]. Reports from Australia [10], Italy [11], the United States [40], and the Untied Kingdom [50] suggested that the lack of sufficient PPE stocks for primary care providers was emblematic of the inadequate consideration of primary care in pandemic response planning.

Study results suggest steps to improve pandemic preparedness. Rapidly evolving guidelines complicated FPs’ efforts to acquire recommended PPE and implement infection prevention and control protocols [5, 6, 8, 20]. Further research is needed to evaluate the various guidelines produced during different stages of the COVID-19 pandemic and identify the types of PPE that are best-suited for primary care in the early stages of a pandemic when little is known about a novel virus to ensure adequate stockpiles can be maintained in preparation for future pandemics. Moreover, given the importance of PPE to maintaining the availability of routine primary care and reducing the stress and anxiety of primary care providers, health systems need to strengthen supply chains, improve stockpile management, and update PPE allocation methods to include the needs of community-based primary care providers.

## Limitations

We interviewed FPs in four regions in Canada between October 2020 and June 2021. Findings may not reflect the experiences of other primary care professionals, those in other areas, or at later stages of the COVID-19 pandemic. Moreover, the majority of participants were paid by alternate payment plans and had hospital affiliations, unlike most FPs in Canada, so findings may under-represent the experiences of fee-for-service and unaffiliated FPs. Interviews, like all self-reported data, are subject to recall and social desirability bias [51, 52].

## Conclusions

FPs in or associated with hospital and health authority-affiliated clinics in Canada had better access to PPE than those in privately-owned community-based practices who often relied on donated, reused, and non-medical grade PPE supplies. PPE shortages heightened fear and anxiety among primary care providers and symbolized the neglect and lack of consideration for primary care in the pandemic response. Study findings highlight the need to balance the risks and needs of hospital and community-based care in PPE conservation and allocation strategies and the influence of the underlying organization of primary care on PPE distribution during the pandemic.

## Supplementary Information

**Additional file 1.** Appendix A. Interview Guide.

## Data Availability

The datasets generated and/or analysed during the current study are not publicly available due to ethical and confidentiality reasons, but some portion of the de-identified data may be available from the corresponding author on reasonable request.

## Abbreviations

PPE: Personal protective equipment
FPs: Family physicians
BC: British Columbia
NL: Newfoundland and Labrador
NS: Nova Scotia
ON: Ontario
